# Physiological Hypoxia (Physioxia) Impairs the Early Adhesion of Single Lymphoma Cell to Marrow Stromal Cell and Extracellular Matrix. Optical Tweezers Study

**DOI:** 10.3390/ijms19071880

**Published:** 2018-06-26

**Authors:** Kamila Duś-Szachniewicz, Sławomir Drobczyński, Piotr Ziółkowski, Paweł Kołodziej, Kinga M. Walaszek, Aleksandra K. Korzeniewska, Anil Agrawal, Piotr Kupczyk, Marta Woźniak

**Affiliations:** 1 Department of Pathology, Wrocław Medical University, Marcinkowskiego 1, 50-368 Wrocław, Poland; ziolkows@interia.pl (P.Z.); k.m.walaszek@gmail.com (K.M.W.); piotr.kupczyk.81@o2.pl (P.K.); marta1wozniak@wp.pl (M.W.); 2 Department of Optics and Photonics, Wrocław University of Science and Technology, Faculty of Fundamental Problems of Technology, Wybrzeże Wyspiańskiego 27, 50-370 Wrocław, Poland; slawomir.drobczynski@pwr.edu.pl (S.D.); ak.korzeniewska@gmail.com (A.K.K.); 3 Division of Pathology, Sokołowski Hospital Wałbrzych, Sokołowskiego 4, 58-309 Wałbrzych, Poland; hp2010@wp.pl; 4 2nd Department of General and Oncological Surgery, Wrocław Medical University, Borowska 213, 50-556 Wrocław, Poland; dranilpreeti@gmail.com

**Keywords:** cell-to-cell interactions, physiological hypoxia (physioxia), optical tweezers, single cell adhesion, diffuse large B-cell lymphoma (DLBCL), mesenchymal stromal cells (MSC), integrins

## Abstract

Adhesion is critical for the maintenance of cellular structures as well as intercellular communication, and its dysfunction occurs prevalently during cancer progression. Recently, a growing number of studies indicated the ability of oxygen to regulate adhesion molecules expression, however, the influence of physiological hypoxia (physioxia) on cell adhesion remains elusive. Thus, here we aimed: (i) to develop an optical tweezers based assay to precisely evaluate single diffuse large B-cell lymphoma (DLBCL) cell adhesion to neighbor cells (mesenchymal stromal cells) and extracellular matrix (Matrigel) under normoxia and physioxia; and, (ii) to explore the role of integrins in adhesion of single lymphoma cell. We identified the pronouncedly reduced adhesive properties of lymphoma cell lines and primary lymphocytes B under physioxia to both stromal cells and Matrigel. Corresponding effects were shown in bulk adhesion assays. Then we emphasized that impaired β1, β2 integrins, and cadherin-2 expression, studied by confocal microscopy, account for reduction in lymphocyte adhesion in physioxia. Additionally, the blockade studies conducted with anti-integrin antibodies have revealed the critical role of integrins in lymphoma adhesion. To summarize, the presented approach allows for precise confirmation of the changes in single cell adhesion properties provoked by physiological hypoxia. Thus, our findings reveal an unprecedented role of using physiologically relevant oxygen conditioning and single cell adhesion approaches when investigating tumor adhesion in vitro.

## 1. Introduction

Diffuse large B-cell lymphoma (DLBCL) is the most common subtype of non-Hodgkin lymphomas (NHL) and it constitutes approximately a third of all NHL [1]. Common features of NHLs is the bone marrow involvement, for DLBCL it concerns up to 40% of cases [2]. Accumulating evidences confirm the critical role of cellular interactions between cancer cells and bone marrow or/and lymphatic tissue microenvironments in lymphoma growth, chemoresistance [3], and survival [4] through a combination of adhesion molecules, chemokines, and cytokines [5,6,7,8].

Most of the experimental research exploring lymphoma-mesenchymal cells interactions has presented the average properties of bulk samples of cells. In this paper, using a panel of human diffuse large B-cell lymphoma cell lines, we characterized the influence of environmental physiological hypoxia on both: single cancer-stromal cell and cancer-microenvironment interactions using optical tweezers. Additionally, the oxygen-related changes in the early adhesion of the primary lymphocytes B in the context of a living cells in their microenvironment have been explored. 

At the cellular level, in vivo oxygen is known to play a critical role in the regulation of a variety of processes guiding the cell survival and metabolism [9,10]. Generally, in vitro experiments are performed under atmospheric oxygen environment, which are highly non-physiological conditions [11]. The increased oxidative metabolism results in the disturbed production of reactive oxygen species (ROS) and the induction of oxidative stress [12]. Consequently, elevated oxygen regulates cell activity from the gene [13] to the proteome level [14], thus inducing extensive phenotype changes [15,16,17]. Previous studies demonstrated that activity of mesenchymal stromal cells is strongly governed by local microenvironment, where O_2_ level is a critical factor [18]. However, the role of oxygen concentration in cell-to-cell adhesion remains unclear. 

Cell adhesion changes, which are critical for cancer cells invasion and metastasis, are mostly studied by bulk adhesion assays on entire cell population. Bulk adhesion assays have proven important in the understanding of mechanical interactions of cells with their environment providing representative data for the entire cell population. Bulk assays allow for the fast generation of statistically significant average data for large number of cells, however they do not provide the detailed information on single cell behavior. Consequently, small differences in cell adhesion with expected biological relevance are practically undetectable. Meanwhile, cells, even from the same established cell line, are individuals and can differently react to external stimuli and environmental changes. Moreover, cultured cells can expose heterogeneous populations with very different adhesive behavior were even cultured and treated in exactly the same way. Thus, to identify outliers from the main population or analyse the rare cell types, the single cell adhesion assays are required. Additionally, single cell adhesion techniques provide better control over a specific adhesive interaction from the non-specific interactions that can influence the overall cell adhesion. To address this, we need to directly analyze in real-time the interactions between individual living cell and its microenvironment. Single cell adhesion approaches have high potential for understanding how cells regulates adhesion in physiological and pathological conditions.

Individual living cell selection and manipulations have now been made possible by e.g., optical tweezers (OT) [19]. The technology of optical tweezers is constantly developing within life sciences and many applications for study cell migration [20,21], adhesion [22,23,24], tissue remodeling [25,26], and localized hyperthermia [27] have been demonstrated. In contrast to bulk adhesion assays, OT enables to select cell subpopulation with distinct adhesion properties as well as to precisely control short contact times of nascent adhesion formation. The presented here optical tweezers setup was previously used by our group to study adhesion properties of the lymphoma cancer cells [28]. The measurements were performed with HS-5 stromal cell line and the SUDHL-10 lymphoma cell line, as followed: two 3 μm streptavidin coated polystyrene beads were catch by multitrap system and contact with the surface of individual biotinylated lymphoma cells. After a fixed time, until a stable connection was established, the trapped bead was pulled in order to measure the adhesion forces in the pico-newton range. The current approach that is presented in this study eliminates the introduction of beads to the experimental setup and cell surface protein biotinylation, which significantly influences the cell adhesive properties [29,30]. Furthermore, the measurement itself using the two polystyrene beads that are attached to the cell is more time-consuming and complex, as evidenced by much of papers in which maximum 20–30 cells were analyzed. Herein, the authors were interested in finding the effective method for single cell adhesion testing of the largest possible statistical probe. We expected that the initiation of cell-to-cell/cell-to-extracellular matrix adhesion and detection the adhesion changes from external stimuli (e.g., physioxic treatment) at time-scale has the potential to operate at high throughput rates.

Cell adhesion is mediated by cell surface receptor macromolecules, including integrins, cadherins, selectins, and members of the immunoglobulin superfamily. Those proteins can specifically bind: either the molecules of the extracellular matrix (ECM) or receptor molecules of other cells. The proteins mostly responsible for early adhesion processes are integrins. Integrins are heterodimeric cell surface receptors expressed on most human cells where they mediate cell-cell and cell-extracellular matrix interactions [31]. It is widely accepted that the deregulation of integrins results in adhesion changes in several solid and hematological cancers, including acute leukemia [32,33], chronic leukemia [34], or multiple myeloma [35]. While several results concerning severe hypoxia in solid tumors indicates the overregulation of integrins [36,37,38], the impact of physiological hypoxia on their expression among lymphomas is poorly investigated. 

To address above issues, we aimed to characterize the interactions between lymphoma and mesenchymal stromal cells in normoxic and physioxic conditions in time-scale using optical tweezers and population adhesion assays. Our study represents a significant advance over previous systems that were employed to study B-cell-stromal cell and B-cell-microenvironment interactions. These typically involved quantifying the amount of attachment of cells over a period of >30 min, which was incompatible with rapid formation of nascent adhesion. The implementation of optical tweezers allowed for precisely controlling the initiation of cell-cell contact following by termination of interactions at a defined time point. In our experimental setup, the reduced oxygen environment (from atmospheric to a more physiological level-5%) affected both: lymphoma and primary B-cells adhesion to MSCs and extracellular matrix (Matrigel). Concurrently, adhesion changes were found to be fully reversible after 72 h of reoxidation. Thus, we conclude that culturing DLBCL cells at physioxic oxygen concentration simulate physiological environment, resulting in cell behaviors that are much more closer to the tumor microenvironment. Therefore, precise oxygen concentration should be considered when designing and performing experiments with DLBCL cells. Data obtained in this study underline the necessity of validating outcomes from adhesion studies that are performed with lymphoma cell culture under ambient O_2_.

## 2. Results

### 2.1. DLBCL Cell Lines Exhibit Differential Proliferative Response to Experimental Physioxia and Hypoxia

**Figure 1:**
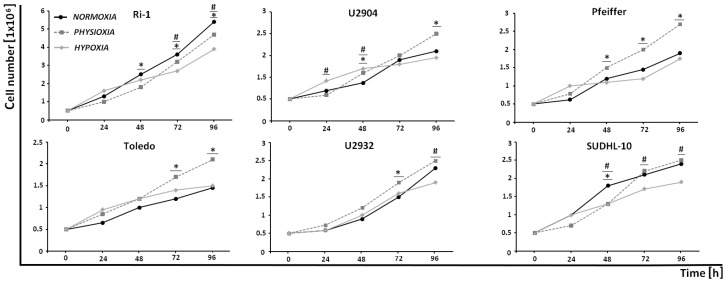
Cell growth of six lymphoma cell lines under hypoxia (1% O_2_), physioxia (5% O_2_), and normoxia (21% O_2_). Evaluations were made at 24, 48, 72, and 96 h from the beginning of the incubation under various oxygen conditions. Representative data are from at least 2 parallels from three independent investigations. The symbol (*) indicates a significant difference between incubation in physioxia and normoxia considering a *p*-value <0.05, while symbol (#) indicates a significant difference between incubation in hypoxia and normoxia while considering a *p*-value <0.05; Student’s *t*-test.

To determine the effect of physioxic (5% O_2_) and hypoxic (1% O_2_) treatment on DLBCL cell lines proliferation, we measured cell viability by Trypan blue test. Representative graphs are shown in Figure 1 for six DLBCL cell lines.

No differences were detected between lymphoma cells proliferation after 24 h of physioxic and hypoxic treatment, except the U2904 cell line. In turn, hypoxia modestly decreased the proliferation of Ri-1, U2932, and SUDHL-10 after 96 h, while the proliferative capability of the remaining cell lines was unchanged. The cessation in growth of DLBCL cell lines under hypoxic stress was recently described by Bhalla et al. [15]. Regarding physioxic treatment, while the growth of U2904, Pfeiffer, and Toledo cell lines significantly increased after 96 h of incubation, for U2932 and SUDHL-10 cells no differences in proliferation rates were observed. At the same time the Ri-1 cells only exhibited decreased growth in physioxia. Together, in vitro growth data suggests that the effect of physioxic and hypoxic treatment on DLBCL proliferation strickly depends on the lymphoma cell type. 

### 2.2. Hypoxia-Inducible Factor 1-Alpha Expression is Altered in Physioxia

**Figure 2:**
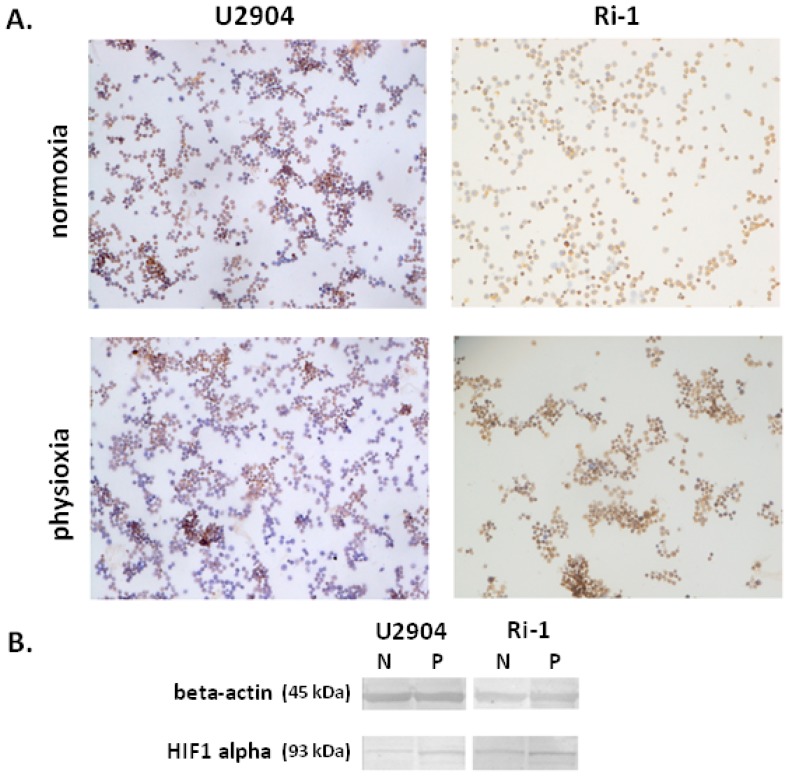
Immunocytochemical (**A**) and Western blot analysis (**B**) of the hypoxia-inducible factor 1-alpha (HIF1α) expression in lymphoma cell lines U2904 and Ri-1 under normoxia (21% O_2_) and physioxia (5% O_2_). Magnification ×200.

B-cells in the body are exposed to varying oxygen concentration, which is significantly lower than atmospheric oxygen environment. One of the most important transcription factor that mediates the cellular responses to low oxygen environments is hypoxia-inducible factor 1-alpha (HIF1α). The role of HIF1α has been deeply investigated in the context of severe hypoxic stresses (<1% O_2_), however its induction under physioxia remain elusive. Here, we determined if the expression of HIF1α protein was altered under physioxic conditions in Ri-1 and U2904 cell lines. Lymphoma cells were grown at 21% and 5% O_2_ for 48 h to determine HIF1α levels in respective cell lines by immunocytochemical and Western blot analysis. Altered activation of HIF1α protein was confirmed in both cell lines in physioxic conditions when compared with cells that were cultured at 21% oxygen (Figure 2).

However, the expression of HIF1α that was observed in our study in physioxia was much more reduced than those that were recently established by Bhalla et al. at hypoxia (1% O_2_) on the panel of DLBCL cell lines [15]. Concurrently similar observations regarding HIF1α induction in physioxia were previously made by Carrera et al. on breast and colorectal cancer cell lines [39].

### 2.3. The Influence of Laser Beam on Living Cells

**Figure 3:**
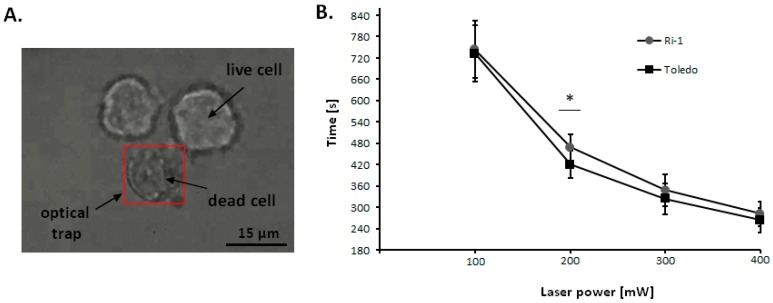
Trypan blue accumulation on the surface of untreated living Ri-1 cells, while dead cell was held in optical trap >300 s at 300 mV of laser power. The red frame indicates the area of operating range of the optical trap, while the focused laser beam is located in the center of trapped specimen (**A**). Characterization of cell death under varied laser power using Trypan blue for Ri-1 and Toledo cell lines in optical tweezers. The measurements were repeated for 10 individual cells for each laser power. The symbol (*) indicates a significant difference in cell death between Ri-1 and Toledo cells considering a *p*-value < 0.05; Student’s *t*-test (**B**).

Optical trapping of eucaryotic cell May possibly induce photodamage, which is influenced mainly by the time of exposure, the laser power, and the type of cell [19,24,25]. Here, we used optical tweezers with increasing lasers powers to evaluate the influence of laser beam on Ri-1 and Toledo cells destruction (Figure 3A). The fastes cell death (after 282 s and 264 s of cell trapping for Ri-1 and Toledo cells, respectively) occurred at 100% laser power corresponding to 400 mW (Figure 3B). Statistically significant differences in photosensitivity (*p* < 0.05) was observed between Toledo and Ri-1 cell lines at 50% of laser power only. We established that cell mortality due to photodamhe decreased with the reduced laser power. To manipulate B-cells in all experiments, 25% of laser power (100 mW) with minimal influence on cell viability was used, while the trapping and moving ability were fully maintained. This setting allows for non-invasive laser exposure over 420 s, which was the maximum manipulation time on individual cell in this study.

### 2.4. Single Cell Adhesion in Optical Tweezers

We introduced an optical tweezers technology for testing single B-cell interactions with stromal cell and extracellular matrix in normoxia and physioxia. Stromal cells are essential element of bone marrow microenvironment are an important support of lymphoma cell survival. In this study, we used the stromal cells in order to observe lymphoma-neighboring cells interactions. As a model of ECM, we chose to examine adhesion to Matrigel, which is a complex multiprotein matrix that serves as a more physiological component. 

#### 2.4.1. Varying Single Cell Adhesion to MSC and Matrigel among DLBCL Cell Lines

First, we established that the average time of nascent adhesion formation to HS-5 was from 15.5 ± 8.4 s to 132.9 ± 48.8 s for Ri-1 and Toledo cells, respectively (Table 1). Based on precisely determined contact time values, we were able to distinguish cell lines with high and medium adhesive properties (Ri-1, SUDHL-10, U2932, and Pfeiffer), and the cell lines where evident lower adhesion properties were observed (U2904 and Toledo). Adhesion properties were evaluated for passage 3, 6, and 10, and were found to be stable for each cell line. These observations were numerous and reproducible.

Regarding interactions with Matrigel, cells were trapped for significantly longer in order to form stable connection (from 43.8 ± 14.1 s for SUDHL-10 to 291.2 ± 41.8 s for U2904 cells). Obtained results are in line with nature of cell-cell and cell-microenvironment interactions and certain differences between them. While on the cell surface, hundreds of adhesion molecules and ion channels are able to interact with proteins that were expressed on the adjacent cell, the ability of cell binding to extracellular matrix is mainly mediated by limited number of ECM proteins, including e.g., fibronectin, laminin, collagen (type I and IV), and vitronectin. 

#### 2.4.2. Physioxia Decreased Single Lymphoma Cell Adhesion to Stromal Cell and Matrigel 

**Figure 4:**
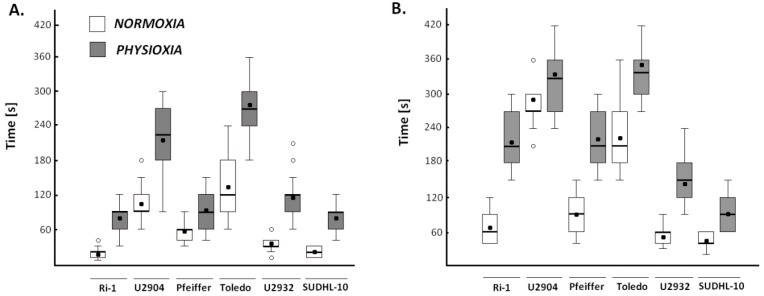
Box and whiskers plot of adhesion changes of single lymphoma cells to mesenchymal stromal cells (**A**) and Matrigel (**B**) when exposed to physioxia for 96 h. A significant difference between single lymphoma cells adhesion in normoxia and physioxia considering a *p*-value < 0.001 were observed for all cases except U2904 cells adhesion to Matrigel. The symbol (°) indicates outliers; Student’s *t*-test.

Then, we asked whether physiological oxygen environment influences adhesion of single B-cell to marrow stromal cell and Matrigel. Figure 4 and Table 2 show the adhesion changes of single lymphoma cell after incubation of 96 h in physioxia. 

We observed that the time required to form a stable connection between lymphoma and stromal cells in physioxia increased from 1.7 up to 5.1 times for Pfeiffer and Ri-1 cell lines, respectively (Table 2). Regarding the adhesion of lymphoma cells to Matrigel, the observed fold change was from 1.1 for U2904 cell line to 3.2 for Ri-1 cells, thus cell interactions with Matrigel were less variable under physioxia than interactions with stromal cells. Nevertheless, the Ri-1 cell line was found to be the most sensitive to hypoxia, while the U2904 cell line was the most resistant. All of this data indicates lower adhesive properties of lymphoma single cell to both: adjacent cells and extracellular matrix in physioxia, however, the rate of cellular response to hypoxia varies among cell lines.

#### 2.4.3. Physioxia Decreased Primary B-Cell Adhesion to Stromal Cell

**Figure 5:**
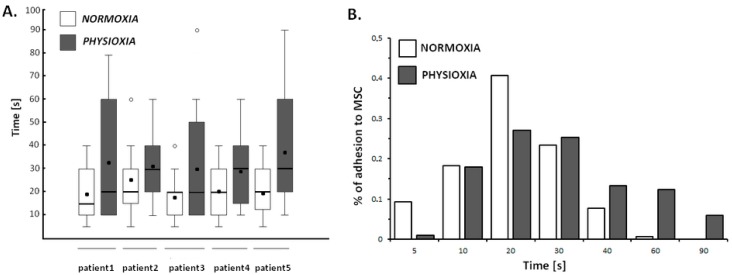
Box and whiskers plot of time-dependent adhesion of primary B-cells isolated from lymph node to marrow stromal cells in normoxia and physioxia. A significant difference between primary B-cells adhesion in normoxia and physioxia while considering a *p*-value < 0.01 was observed for all cases. The symbol (°) indicates outliers; Student’s *t*-test. *N* = 60 for each patient in normoxia and physioxia (**A**). The distribution of time-dependent adhesion to MSC in normoxia and physioxia (**B**).

Prior to optical tweezers manipulations, primary B-cells were isolated from surgically removed lymph nodes, according to the protocol described in the Material and Methods section. The effect of physioxic treatment on single primary B-cell adhesion to stromal cell was evaluated according protocol established for cell lines. In total, adhesion of 600 primary cells obtained from five patients was analyzed at single cell level. We established that primary B-cells adhered to stromal cells within 20.3 ± 10.3 s in normoxia vs. 32.8 ± 22.8 s when exposed for 24 h on physioxia. The Figure 5A shows that lymphocytes under physiological oxygen conditions showed significantly greater variability in adhesion properties than the normoxic cells. 

Interestingly, while 9.3% of normoxic cells adhered to stromal cells within 5 s, only 1% of physioxic cells established stabile bond to MSCs during this time (Figure 5B). Concurrently, the maximum adhesion time of 0.6% of primary B-cells to mesenchymal stromal cells in normoxia was 60 s, the 12.3% and 6% of cells growing under physioxia required 60 s and 90 s, respectively, to form stabile connection between two cell types. 

### 2.5. Cell Adhesion for Entire Lymphoma Population Does Not Reflect Results from Single Cell Assay

**Figure 6:**
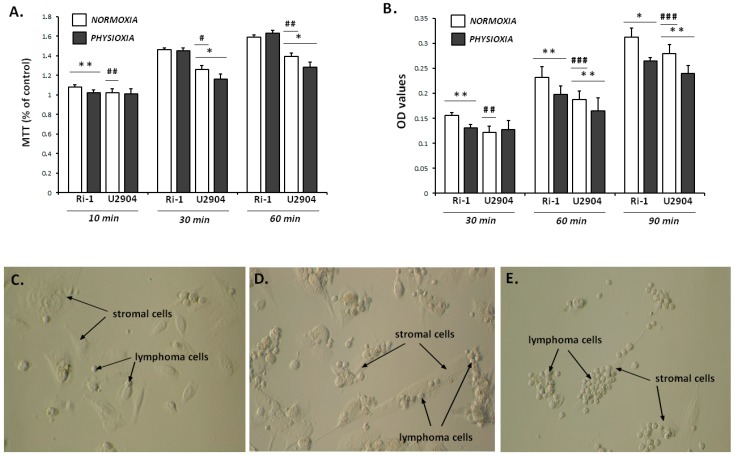
Adhesion of Ri-1 and U2904 cells to mesenchymal stromal cells (**A**) and Matrigel (**B**) in normoxia and physioxia. Each column represents the average of three independent replicates. Error bars represent ± S.D. The symbols (*) and (**) indicate a significant differences in lymphoma cells adhesion in normoxia and physioxia considering a *p*-value < 0.05 and <0.01, respectively. The symbols (^#^), (^##^) and (^###^) indicate a significant differences between Ri-1 and U2904 cells adhesion in normoxia considering a *p*-value < 0.05, <0.01 and <0.001, respectively; Student’s *t*-test. Ri-1 cells adhesion to stromal cells after 10 min (**C**) and 30 min (**D**) of co-incubation. Toledo cells line adhesion to stromal cells after 60 min of co-incubation (**E**). Magnification ×200.

Out of several commonly used bulk assays to study cell adhesion, the washing assay is the most frequently used one. In brief, in this method, cells are seeded onto an adhesive surface, allowed to adhere for a given time, followed by washing with physiological buffer. As a result, non or weakly attached cells are detached from the adhesive substrate and the remaining attached cells are determined. In this study, we exposed representative Ri-1 and U2904 cell lines for physioxia (96 h), followed by the determination of adhesion of entire cell population to stromal cells and Matrigel. We noted that lymphoma cell lines differ in the percentages of adhesion to mesenchymal stromal cells after 30 and 60 min of co-incubation (Figure 6A). The maximal adherence to stromal cells occurred within 60 min of co-incubation for Ri-1 and Toledo cell lines. The results showed no differences in Ri-1 cell adhesion in bulky test after physioxic treatment when compared with normoxia, however, significant reduction in the number of U2904 cells attached to stromal cells after 30 and 60 min was observed. Thus, the adhesion of U2904 cells to mesenchymal stromal cells was significantly suppressed. Lymphoma cells-to-MSCs adhesion in is presented in Figure 6C,D).

Regarding the adhesion to extracellular matrix, physioxic treatment diminished Ri-1 cells adhesion to Matrigel in each time point, whereas the adhesion of U2904 cells after 30 min of incubation remained unchanged (Figure 6B). Interestingly, U2904 cells adhered slower both to stromal cells and Matrigel when comparing to Ri-1 cells, what is in agreement with our optical tweezers manipulations on a single cell level. Additionally, we observed that lymphoma cell lines adhere faster to stromal cells than to Matrigel, both in normoxia and hypoxia. However, based on our investigation, the differences in cell adhesion between cell lines in bulky assay are not as relevant as our observations from single cell assay. In particular, with a bulky test, we were not able to precisely establish neither the adhesion profile of lymphoma cell lines nor the role of oxygen influence on cellular adhesion as we did by performing optical tweezers manipulations.

### 2.6. Adhesion Changes under Physioxia Are Fully Reversible

We decided to investigate whether the decrease in cell-to-cell adhesion that was observed under physioxic treatment is reversible and analyze the dynamic of reoxygenation. For this purpose, representative cell lines were incubated for 96 h in physioxia, following by single lymphoma cell adhesion to mesenchymal stromal cells assessment in time-scale in optical tweezers after 24, 48, 72, and 96 h of cell incubation in normoxia.

**Figure 7:**
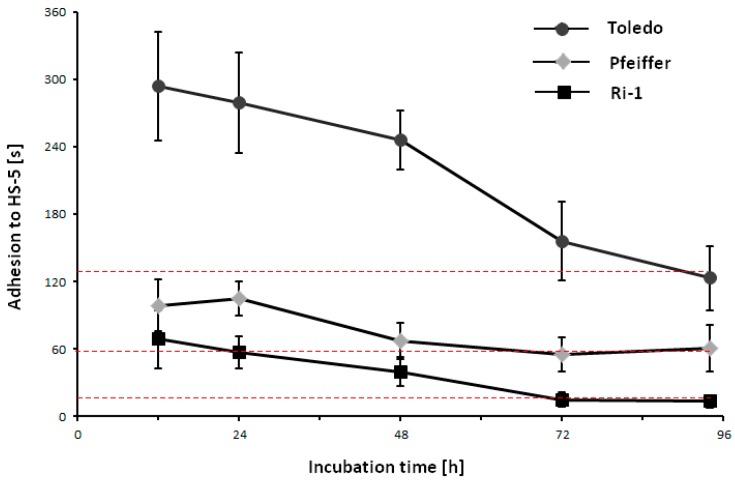
Adhesion changes of single lymphoma cells to mesenchymal stromal cells evaluated in optical tweezers in time-scale for Ri-1, Pfeiffer, and Toledo cells during the reoxydation process. Evaluations were made at 24, 48, 72, and 96 h from the beginning of the cell incubation in normoxia. Data are representative of 10 measurements for each cell line at each time point ± S.D. Red dotted lines indicate the average contact time required for the formation of adhesion between lymphoma cells and mesenchymal stromal cells which is 15.5, 55.9, and 132.9 s for Ri-1, Pfeiffer, and Toledo cells, respectively.

We establish that long physioxic treatment of lymphoma cells is reversible if it is followed by 72 h of incubation for Pfeiffer and Ri-1 cell line and 96 h for Toledo cell line in 21% oxygen (Figure 7). In our setup, the dynamics of reoxydation depend strictly on individual cell line properties. After 24 h of reoxydation, we did not observe any significant changes in cell adhesion for any cell line used in the study. For Pfeiffer cell line the largest changes in adhesiveness were observed after 48 h of reoxydation, while for Ri-1 and Toledo cell lines it occurred after 72 h. 

### 2.7. Anti-ITGB1 and Anti-CDH2 Treatment Caused Significant Decrease in Single Lymphoma Adhesion 

**Figure 8:**
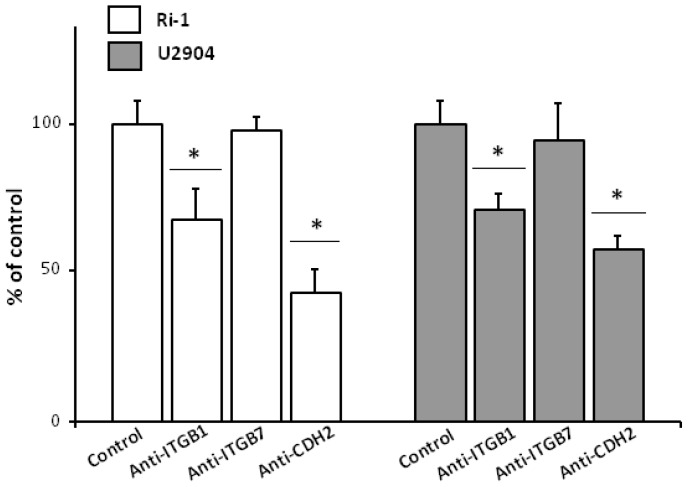
The adhesion molecules blocking study evaluated in optical tweezers. The examined cellular adhesion molecules (CAMs) are integrin β1 (ITGB1), integrin β7 (ITGB7), and cadherin-2 (CDH2). The percentage of lymphoma cells that stable bond to stromal cells within 40 and 300 s were calculated for Ri-1 and U2904 cell line, respectively. Data are expressed as mean  ±  SEM in tree independent experiments for 30 cells for each experimental condition. The symbol (*) indicates a significant difference in single cell adhesion after representative antibody treatment when compared to untreated cells considering a *p*-value < 0.001; Student’s *t*-test.

In order to evaluate potential effect of blocking β1 and β7 integrins and cadherin-2 on lymphoma cell adhesion, we treated Ri-1 and U2904 cell lines with representative antibodies being recommended for blocking assays. The effects of antibody blocking on lymphoma adhesion to stromal cells were evaluated in optical tweezers using the above described setup. For Ri-1 and U2904 cells, we observed significantly decreased adhesion to stromal cells after treatment with anti-ITGB1 and anti-CDH2 antibodies, whereas no differences were found for anti-ITGB7 antibody (Figure 8). 

The largest decrease in percentage of the adhesive cells for both Ri-1 and U2904 was observed for cadherin blocking, which suggests that those proteins are essential for lymphoma adhesion. Altogether, these results show that integrin β1 and cadherin-2 are required for lymphoma-stromal cell interactions and could partially account for the decrease of adhesion under physioxia precisely validated in time-scale in optical tweezers in this study. 

### 2.8. Physioxia Impaired Integrin β1, β2 and Cadherin-2 Expression

**Figure 9:**
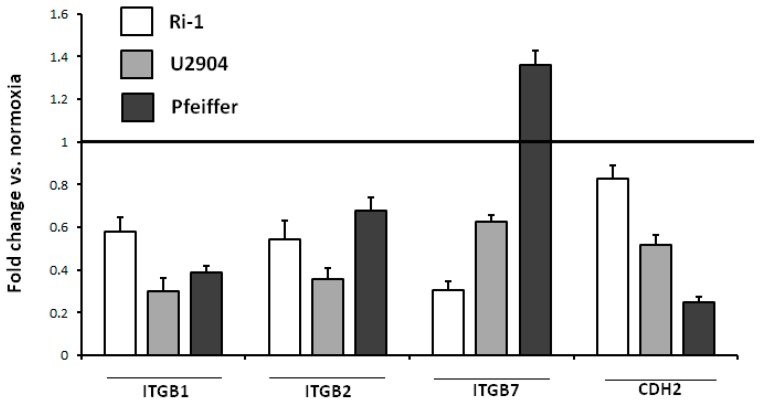
Fold change in cellular adhesion molecules expression for Ri-1, U2904, and Pfeiffer cell lines after incubation of 72 h in physioxia examined by confocal microscopy. Evaluated cellular adhesion molecules (CAMs) are: integrin β1 (ITGB1), integrin β2 (ITGB2), integrin β7 (ITGB7), and cadherin-2 (CDH2).

To further confirm that observed adhesion lost in contact with stromal cells observed in physioxic conditions could be partially integrins β1, β2, β7, and cadherin-2 dependent, the expression changes of these CAMs in normoxia and physioxia were evaluated by confocal microscopy. Representative data for Ri-1, U2904, and Pfeiffer cell lines are shown in Figure 9. 

**Figure 10:**
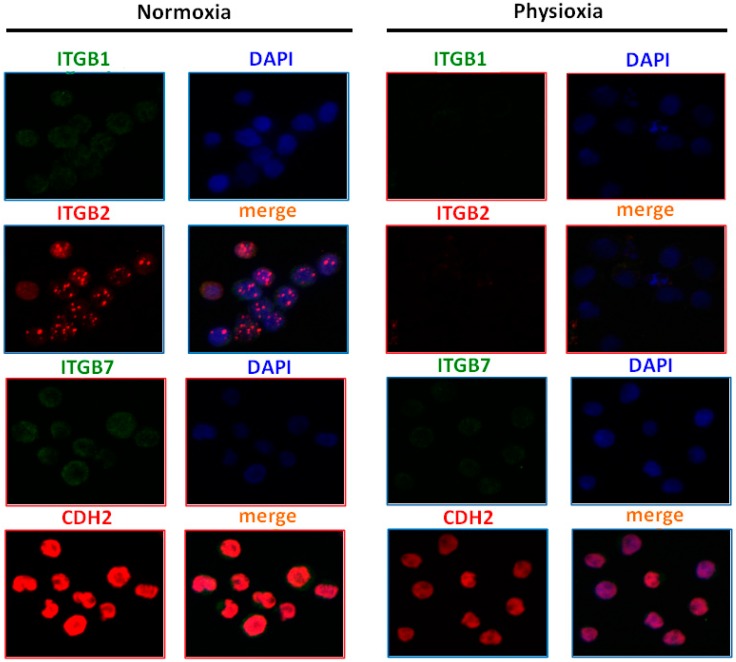
Confocal microscopy images of U2904 cells expressing cellular adhesion molecules (CAMs) under normoxia and physioxia. Cells were preincubated in normoxia and physioxia for 72 h and stained with anti-ITGB1, anti-ITGB2, anti-ITGB7, anti-CDH2, and a nuclei probe (DAPI). Magnification at 63× and zoom in.

As evidenced by confocal microscopy, the expression of integrins β1 and β2 as well as cadherin-2 was significantly impaired for all cell lines after physioxic exposure for 96 h (Figure 10). Integrin β1 was the protein that was most affected by oxygen decrease, while U2904 was the cell line where the biggest changes in cellular adhesion molecules expression were detected. Interestingly, in Pfeiffer cells, we observed a 35% (1.3 fold) increase in the expression of integrin β7 under physioxia. Taking into account no effect of integrin β7 blocking on lymphoma adhesion, we could emphasize the marginal role of this protein for lymphoma adhesion in comparison to β1 and β2 integrins.

## 3. Discussion

In the cell culturing, scientists are making an effort to control the cellular environment to better mimicking in vitro conditions. For instance, in human cell culture cells are grown at 37 °C and in the presence of 5% CO_2_ to imitate physiological temperature and pH, respectively. Surprisingly, oxygen concentration is a forgotten parameter. However, in the last 10 years, the emerging evidence confirms that culturing cells in ambient air (normoxia) is far from physiological, because most tissues do not exhibit such high oxygen concentrations [40]. Oxygen level in tissues (physioxia and physiologic hypoxia), ranging 3–10% (23–70 mmHg), is therefore considered as physioxic in respect to atmospheric air [41]. In turn, the hypoxia usually observed in solid tumors is indicative of a deficit of oxygenation in tissues. 

Culturing cells in physioxia is not a new concept [42], but it has not received approval for many years because cells that are cultured in ambient air have historically grown quite well. However, recently observed rapid development of three-dimensional (3D) cancer models re-emphasizes the need to mimic the tumor microenvironment, including the oxygen tension, which has been considered as the limiting nutrient for 3D cell cultures [43,44]. Another important research trend on physioxia observed lately is human fibroblast [45] and steam cells biology [46,47,48].

When considering the contributions from oxygen in cell culture and arising questions whether normoxia could change/interfere with physiological phenotype, we investigated how the reduction of environmental oxygen that is relevant to tissue physiological concentration affects single lymphoma and primary lymphocyte B adhesion in the context of a living cells in their microenvironment.

Generally, cell adhesion assays could be grouped into the cell population and single cell approaches, and the use of a certain method strictly depends on cell’s specific applications, e.g., studies of cell signaling, drug treatment, biomechanics, tissue engineering, etc. In this work, we decided to implement the optical tweezers manipulations [49] to precisely characterize the adhesion profile of six human DLBCL cell lines and primary lymphocytes B at the single cell level. To the authors’ knowledge, indeed, the research on lymphoma-stromal cells/extracellular matrix interactions at the single cell level was carried out for the first time. Previously published studies used the optical tweezers to determine leukemic cells-stromal cell interactions by measuring nanoforces between them [24,50,51]. However, during pilot study, we observed that, after stabile connection between two cell types (e.g., B-cell and MSC) was formed, we were not able to separate them by the use of laser at maximum power of 400 mW. Although OT are the only technique able to provide contactless manipulations on individual organelles and molecules without damaging cell wall, their influence on cell viability strictly depends on laser power used in the study. According to the literature and own experience, the application of laser power higher than 400 mW provoked the photodamage shortly [19,52]. For the above reasons, we have decided to implement the capability of OT to contactless moving of microobjects and laser-dependent maintenance of cell-to-cell and cell-to-ECM contact to develop a single cell adhesion assay. Additionally, we aimed to avoid the introduction of biotin and beads to the experimental setup in order to not affect the cell adhesion. 

The complexity of cellular systems requires that an adequate large number of cells be analyzed to derive statistically relevant results. Using the introduced methodology, we analyzed from 25 to 60 cells within single experiment lasting approximately two hours. In total, over 1600 of B-cells adhered to stromal cells or Matrigel were analyzed one by one in a relatively short period of time. When compared to most adhesion studies, including our previous works, were max 30–40 cells analyzed, here we significantly overcame the low throughput of single cell adhesion experiments.

Additionally, we compared single lymphoma cell adhesion that was evaluated by optical tweezers to adhesion of cell population under static conditions (washing assays), and saw the significant variance in the obtained results with potential biological significance. For instance, when bulk adhesion assays typically relay on quantifying the amount of attached cells over a period of >30 min, the formation of nascent adhesion between lymphoma and stromal cell in normoxia is much more rapid (from 10 to 360 s), which has been proven in this work. In relation to physioxic treatment, while there was no effect on B-cell/lymphoma adhesion to stromal cells in population assay observed, we confirmed significant decrease in adhesion in single cell assay. Likewise, adhesion to Matrigel also had little effect in population assays, while there was a significant decrease in adhesion for all analyzed cell lines where single cell manipulation by optical tweezers was applied. Based on our combined investigation, we suggest that single cell approach allows for much more precise determination of changes in adhesion than bulky tests with biological relevance. Additionally, single-cell techniques are required in experiments where population adhesion assays cannot be used because the number of cells that are available for adhesion measurements is limited, e.g., primary cells from biopsy.

Regarding the influence of oxygen concentration on B-cell adhesive properties, our results show that human DLBCL cells as well as primary B-cells exposed to physioxia are less adherent to stromal cells and Matrigel than control cells growing in normoxia. When considering that our work is pioneering in exploring the effects of physioxia on B-cell adhesion, we can only compare our results to research regarding hypoxia. Muz et al. studied the adhesion changes between Waldenström macroglobulinemia cells (non-Hodgkins lymphoma) and bone marrow stroma and confirmed decrease in adhesion after hypoxic treatment [53], which is in agreement with our work. Interestingly, the authors also indicated that the observed decrease in adhesion was mediated by the reduced expression of E-cadherin in hypoxia, as demonstrated by confocal microscopy in our study. An earlier work on adhesion of breast carcinoma cells to the extracellular matrix molecules e.g., vitronectin and fibronectin reported a significant decrease in adhesion after exposure to hypoxia [54]. The observed adhesion decline was reversible by re-exposure to 20% oxygen, which was confirmed in our work. Other bulk adhesion studies have also reported a decrease in cellular adhesion under hypoxia [55,56,57].

Trying to justify the effect of reduction of adhesive properties within B-cells in physioxia, we decided to analyze the integrins and cadherin expression as key players in cellular adhesion [31,58]. Thus, we evaluated the integrin expression on lymphoma cells by fluorescence staining under physioxia and normoxia. Despite the upregulation of β7 integrin in Pfeiffer cell line, we have demonstrated pronounced down-expression of β1 and β2 integrins under physioxia. These changes were accompanied by a decreased level of cadherin expression in all representative cell lines. Importantly, the protein where expression was mostly affected by oxygen decrease was integrin β1, which has the biological relevance because this subunit is mainly responsible for cell-cell and cell-extracellular matrix nascent adhesion [59]. Regarding the results that were obtained from our single cell adhesion study evaluated in optical tweezers, we can emphasize that the decline in integrins is associated with a decrease in adhesiveness of various cancer cells, what is in line with numerous works [54,55,56,60]. Additionally, we confirmed impact of selected CAMs on single B-cell adhesion in optical tweezers by using of function-blocking antibodies to β integrins and cadherin-2 on representative cell lines and primary B-cells. We observed that the formation of adhesion between B-cell and MSC prolonged significantly when β1 integrin and cadherin-2 were blocked with appropriate antibody what is in line with literature [61,62,63]. 

Here, it is needed to emphasize that the present study did not aim at providing further insights into molecular mechanisms of adhesion changes caused by modification of oxygen environment, but was driven by the need to physically confirm differences in lymphoma-stromal cells interactions under physioxia. Currently, further proteomic and metabolomics investigations are being performed to study the molecular background in more detail. To summarize, our study describes for the first time the influence of physiological oxygen on cell-to-cell and cell-ECM adhesion of single lymphoma primary B-cells. Based on this investigation, we suggest that the single cell adhesion assay performed e.g., by optical tweezers allows for much more precise determination of changes in adhesion than population assays. We also emphasize that the characterization of adhesion properties of an individual cell can help in precise and repeatable monitoring of adhesion changes after the cells were treated by external stimuli, including drugs or environmental stressors.

## 4. Materials and Methods 

### 4.1. Cell Lines and Primary Cells

Six human diffuse large B-cell lymphoma (DLBCL) cell lines were used in this study. Pfeiffer and Toledo cell lines were purchased from ATCC (American Type Culture Collection, Manassas, VA, USA), while SUDHL-10, Ri-1, U2904, U2932, and human stromal cell line HS-5 were obtained from Leibniz Institute German Collection of Microorganisms and Cell Cultures (DSMZ, Braunschweig, Germany) were used in this study.

Lymphoma cell lines were cultivated in Gibco™ RPMI 1640 medium with GlutaMax (Thermo Fisher Scientific, Berlin, Germany) containing 10% heat-inactivated fetal bovine serum, FBS (Thermo Fisher Scientific) in a humidified atmosphere. Cultures were maintained by the addition of fresh medium or replacement of medium to maintain cell density, according to the subculturing method for each cell line that was provided by cell supplier. Cell density was measured by Trypan blue assay (Invitrogen Countess Automated Cell Counter, Thermo Fisher Scientific) to maintain cells at a proper density rate. Human mesenchymal stromal cell line HS-5 was cultured in Gibco™ DMEM medium (Thermo Fisher Scientific), supplemented with heat-inactivated 10% fetal bovine serum and 1% Gibco^TM^ l-glutamine (Thermo Fisher Scientific). When cells reached 80% confluence, they were detached with 0.25% trypsin (Sigma-Aldrich, Steinheim am Albuch, Germany) for the following experiments. 

Primary lymphocytes B were obtained from surgically removed reactive lymph nodes for histopathological examination. Written informed consent was obtained from all participants. Lymph nodes were transported in phosphate buffered saline, PBS pH = 7.2 (Thermo Fisher Scientific) with 1% penicillin-streptomycin (Thermo Fisher Scientific) and mechanically dissected by nylon mesh with 70 μm pores (Corning, Wiesbaden, Germany). B-cells were isolated by MACs magnetic isolation using CD20 MicroBeads (Miltenyi Biotech, Bergisch Gladbach, Germany). Cells were centrifuged for 8 min at 1800 rpm at 4 °C, resuspended in RPMI medium, and seeded into a 25 cm^2^ tissue culture flask. The post-MACS B-cells suspension was validated for successful isolation by flow cytometry. B cells were typically 98% pure, as determined by flow cytometry. All of the procedures were performed in accordance with the guidelines and regulations that were set and approved by the local Ethics Commitee of Wrocław Medical University, Poland (KB-504/2014, 1 October 2014). 

### 4.2. Optical Tweezers

**Figure 11:**
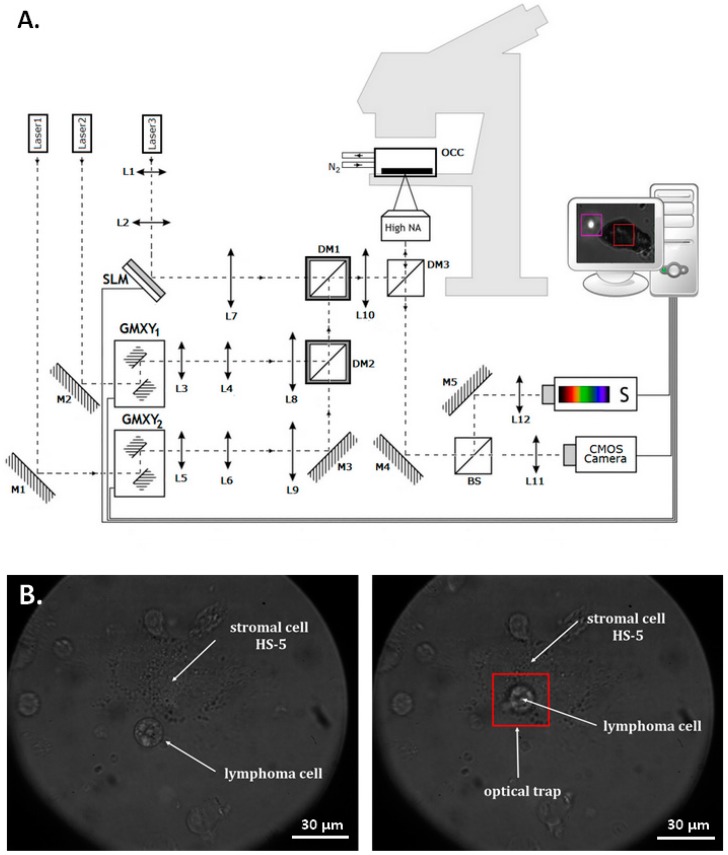
Multifunctional optical tweezers. L1, …, L12-lenses, M1, …, M5-mirrors, DM1, …, DM3-dichroic mirrors, SLM-spatial light modulator, GMXY1,2-Galvano-mirror XY scanning system, BS-beamsplitter, *S*-spectrophotometer, high NA-high numerical aperture objective, OCC-oxygen controlling chamber (**A**). Lymphoma-stromal cell interaction study in optical tweezers (**B**). Toledo cell held in optical trap (**left**) following by assembling to the marrow stromal cell (**right**). The red frame indicates the area of operating range of the optical trap, while the focused laser beam is located in the center of trapped specimen.

A multifunctional optical tweezers instrument was designed and established in the Optical Manipulation Laboratory at the Wrocław University of Science and Technology, Poland. The system has already been used for cellular research e.g., adhesion [28], hyperthermia [27] and biomechanics [64]. Figure 11 shows a scheme of the multifunctional optical tweezers.

In presented setup, optical traps can be generated by three independent lasers differing in wavelength and power. High energy traps are controlled by galvano-mirrors, while the spatial light modulator is used for the creation of different type of traps e.g., Gaussian, Laguerre-Gauss, and Bessel beams by computer-generated-hologram method. All of the manipulations are performed inside an in-house fabricated hypoxic chamber. The physiological oxygen concentration (5%) in the chamber was obtained by pushing atmospheric oxygen through a neutral nitrogen. The entire multifunctional optical tweezers is controlled by home-made software written under C++/CLI for.NET (Microsoft, Warsaw, Poland). The trap stiffness is not the intrinsic property of the optical trap and it depends on both the trap and trapped object. Thus, stiffness measured for one object does not automatically apply to others. The cell has vastly different properties when compared to the polystyrene beads that are commonly used for calibration. Measurements were made using a laser beam with a constant power. It can be considered that the examined cells had a similar morphology, therefore the light-cell interaction was constant. In other words, for constant laser power, the cells were moved with constant force.

### 4.3. Physioxic Conditioning and Reoxidation

Physioxic treatment of lymphoma and stromal cell lines was achieved by incubating cells at 37 °C for 96 h in electronically regulated incubator (New Brunswick Galaxy 48R, Eppendorf, Hamburg, Germany). During incubation, 5% O_2_, 5% CO_2_, and proper humidity were maintained [65]. Primary cells were incubated in 5% O_2_ for 24 h. Controls were preserved 96 h in regular incubator (21% O_2_, 5% CO_2_). To study the reversibility of physioxic treatment, cells were exposed for 96 h to 21% oxygen. 

### 4.4. Cell Viability Assay

To evaluate lymphoma cell viability in 5% and 1% oxygen, Trypan blue exclusion test was performed in 24, 48, 72, and 96 h of cell incubation in normoxic, physioxic, and hypoxic conditions. The evaluation of cell population density and cell viability was performed on the automated cell counter. Living cells were presented as mean percentages of the total cell number ± SD (*n* = 3). HS-5 stromal cells proliferation was assessed with MTT Tetrazolium Assay (Sigma-Aldrich), according to manufacturer instructions. 

### 4.5. The Influence of Laser Beam on Living Cells

2 × 10^4^ of lymphoma cells were add to 10 μL of Trypan blue dye, mixed carefully, and placed onto a glass bottom dish (Greiner bio-one, Frickenhausen, Germany). Single lymphoma cell was trapped in optical tweezers until cell membrane disintegration, followed by dye penetration into cell was observed. The laser power of 100, 200, 300, and 400 mW was tested prior to the selection of the optimal trapping force for living cell manipulations. The experiment was performed on Ri-1 and Toledo cell lines. 

### 4.6. Evaluation of Single Cell Adhesion in Optical Tweezers

The application of optical tweezers to investigate lymphoma-stromal cells interactions at the single cell level has been reported in our previous work [28]. In the method presented here, the B-cells are optically trapped and no exogenous beads are added to the experimental setup. In brief, the glass bottom dish with mature stromal cells was placed inside the chamber mounted on the motorized stage of microscope. For adhesion evaluation in physioxia, the 5% oxygen level inside chamber was maintained.

Adhesion properties of control cells were determined at atmospheric oxygen environment. Briefly, 100 μL of B-cell suspension (1 × 10^5^ cells/mL) was applied directly onto a glass bottom dish with mature HS-5 mesenchymal stromal cells. The time of cell sinking to the glass bottom ranged from 3 to 5 min. Next, as shown in Figure 10B, the cell of interest was individually held in an optical trap (laser power 100 mW) and relocated toward the central part of stromal cell. B-cell was maintained in optical trap to the point at which stable connection with fibroblast was formed. Then, the optical trap was moved away for 15 s, followed by three detach tests to detect whether the B-cell adhered permanently to the MSC. The experimentally established contact time intervals for B-cell trapping were 5, 10, 20, 30, 40, 60, 90, 120, 150, 180, 210, 240, 270, 300, 360, and 420 s, depending on individual cell line properties. Single B-cell was assembled to the stromal cell a maximum of three times and the entire time of individual cell manipulation did not exceed 420 s. The study was performed for each individual lymphoma cell line from passage 3, 6, and 10. For primary lymphocyte B, the three independent experiments for each clinical sample were carried out. 

### 4.7. Adhesion to Matrigel 

Glass bottom dish was coated for 2 h at 37 °C in 2.5 mg/mL Matrigel (Corning, Tewksbury, MA, USA), diluted in serum free RPMI, and washed with PBS. 100 μL of cell suspension (1 × 10^5^ cells/mL) was applied directly onto dish. The time of cell sinking to the glass bottom ranged from 3 to 5 min. Individual B-cell was trapped and assembled to Matrigel until stable connection was formed. The parameters of optical manipulations were preserved from experiments with mesenchymal stromal cells. 

### 4.8. Cell Attachment Bulky Assays

#### 4.8.1. Lymphoma Adherence to Matrigel

96-well plates were coated 2 h at 37 °C in 2.5 mg/mL Matrigel diluted in serum free RPMI and blocked with 1% bovine serum albumin (BSA, Sigma-Aldrich) in PBS. Cells were preincubated for 1 h in serum free RPMI and 5 × 10^5^ cells/well were seeded and incubated 30, 60, 90, and 120 min at 5% and 21% oxygen. Next, nonattached cells were gently rinsed with warm PBS, remaining cells were fixed with 4% paraformaldehyde and stained with 0.1% crystal violet (Sigma-Aldrich). After washing with water, the stained cells were extracted with 0.25 mL of 10% acetic acid (Sigma-Aldrich), and the absorbance of the dye extract was measured at 590/540 nm (ELX800 multi-well reader, Bio Tek Instruments, Winooski, VT, USA). Each assay was performed in triplicate.

#### 4.8.2. Lymphoma Adherence to Mesenchymal Stromal Cells

HS-5 mesenchymal stromal cells were plated in 96 well plates at a density of 8 × 10^4^ cells/well and cultured for 48 h for the confluence 90% in 5% and 21% O_2_. Next, the supernatant was aspirated and counted cell lines U2904, Ri-1, Pfeiffer were plated at a density of 4 × 10^4^ cells/well and incubated in 5% and 21% O_2_ for 10, 30, and 60 min with HS-5 stromal cells. Following each time point, wells were washed three times with warm PBS and the MTT solution was added to the wells in a final concentration of 0.5 mg/mL. After 3 h of incubation at 37 °C, optical absorbance (OA) was measured at 490 nm. The optical absorbance in the control group of HS-5 stromal cells without B-cells was regarded as 100%. The percentage of B-cells stabile adhered to the stromal cells (BS) in each time point (10, 30, 60 min) was calculated according to: BS (%) = (OA of experimental group/OA of control group) × 100. Each assay was performed in triplicate.

### 4.9. Inhibition of Cell Adhesion by Antibodies Targeting Cellular Adhesion Molelecules

For inhibition experiments, representative lymphoma cell lines were pre-incubated with anti-ITGB1 (abx011001, Abbexa, Cambridge, UK), anti-ITGB2 (ab131044, Abcam, Cambridge, UK), anti-ITGB7 (Santa Cruz Biotechnology, Dallas, TX, USA), or anti-CDH2 (HPA046119, Sigma-Aldrich) antibodies for 30 min at 4 °C. The dilution of antibodies was 1:100. Cells treated with goat IgG (ab37373, Abcam) served as control. Next, the cells were centrifuged at 1800 rpm for 10 min at 4 °C, washed twice in PBS, and resuspended in fresh RMPI medium prior to evaluation of adhesion to mesenchymal stromal cells in time-scale in optical tweezers.

The percentage of lymphoma cells that stable bond to stromal cells within 40 and 300 s in optical tweezers were calculated for Ri-1 and U2904 cell line, respectively. Data were expressed as mean  ±  SEM in tree independent experiments for 30 cells for each experimental condition.

### 4.10. Integrin Profile of Lymphoma Cells. Immunofluorescence using Confocal Microscopy

For the immunofluorescence analysis of the integrin profile, Ri-1, U2904, and Pfeiffer cells were cultured in 21% and 5% of oxygen for three days. Following incubation, cells were washed twice in PBS, fixed in 2% formaldehyde (Sigma-Aldrich) for 10 min at room temperature, and adhered to slides by cytocentrifugation at 3000 rpm for 10 min (Cyto-Tek 2500 Cytocentrifuge, Sakura, Alphen aan den Rijn, The Netherlands). After PBS rising, cells were incubated in blocking solution containing 3% BSA, 5% Normal Donkey Serum (Abcam), 0.2% Triton X-100 (Sigma-Aldrich), and 0.05% Tween 20 (Sigma-Aldrich) in PBS for 1 h at 4 °C. Then, the primary antibodies: anti-ITGB1 (1:100, Abbexa), anti-ITGB2 (1:100, Abcam), anti-ITGB7 (1:100, Santa Cruz Biotechnology) and anti-CDH2 (1:100, Sigma-Aldrich) were applied to the slides and incubated overnight at 4 °C. The next day, after PBS washing, cells were incubated with secondary conjugated fluorochrome antibodies: Alexa Fluor 488 anti-rabbit (1:500, H+L, Invitrogen) and Alexa Fluor 555 anti-mouse (1:500, H+L, Invitrogen) at room temperature and darkness conditions for 2 h. After washing, cells were counterstained with DAPI (Abbot, Santa Clara, CA, USA) for nucleus counterstaining. The negative control was performed via omitting the primary antibodies.

The fluorescence scanning-confocal microscopy (Carl Zeiss, Dublin, CA, USA) platform equipped with ×63 objective (Carl Zeiss, USA) and oil immersion was used for cell imaging. The laser wavelengths that were used for excitations was 405 nm for DAPI, 488 nm for Alex Fluor 488, and 568 nm for Alexa Fluor 594 with constant 350 ms time exposition and settings of the camera in all conditions, respectively. All of the obtained z-stacks from four different areas were converted into two-dimensional images and saved as TIFF files using AxioVision Software (Carl Zeiss, USA). Further analysis of semi quantitative fluorescence intensity was performed using ImageJ (Fiji, U. S. National Institute of Health, Bethesda, MD, USA).

### 4.11. Immunocytochemical Analysis of Hypoxia Inducible Factor Alpha

For the immunocytochemistry, cells were prepared as for immunofluorescence analysis. Briefly, after cytocentrifugation cells were washed in PBS, permabilised in 0.1% Tween 20 for 10 min, washed, and incubated with 3% endogenous peroxidase blocking buffer (Abcam). Following incubation with protein blocking buffer (Abcam), primary antibody anti-HIF1α (1:100, Abcam) was applied and slides were stored overnight at 4 °C. The next day, the slides were washed with PBS and incubated for 1 h at room temperature with anti-rabbit secondary antibody (1:1000, Abcam). Then, the slides were rinsed twice with PBS and stained with 3,3’-diaminobenzidine (DAB) in chromogen solution (Sigma-Aldrich). Finally, cells were counterstained with Mayer’s hematoxylin (Sigma-Aldrich), dehydrated in graded alcohols, cleared in xylene, and mounted with xylene-based mounting medium. The negative controls were obtained by omitting the primary antibodies. Photographs were taken by light microscope fitted with a digital camera (Nikon Eclipse 80i with camera DS-Fil-U2, Nikon Instruments, Amsterdam, The Netherlands) at magnifications of 200×.

### 4.12. Western Blot Analysis of HIF1α

5 × 10^5^ cells were washed with PBS and centrifuged at 1800 rpm for 10 min. Cell pellets were lysed with RIPA buffer (Thermo Fisher Scientific) containing protease and 1% phosphatase inhibitors cocktail (Sigma-Aldrich, Germany). The lysates were cleaned by centrifugation at 12,000 rpm for 30 min. The supernatant was collected and the protein concentration was measured using Qubit Protein Assay (Thermo Fisher Scientific). Total protein extracts (50 µg) were separated on 4% to 12% gels SDS-PAGE (sodium dodecyl sulfate polyacrylamide gel electrophoresis, Invitrogen) and transferred to the nitrocellulose (AmershamHybond, Healthcare Bio-sciences AB, Uppsala, Sweden). The membrane was blocked with PBS containing 0.1% Tween 20 with 10% goat serum (Sigma-Aldrich) for 1 h at room temperature. Subsequently, the membrane was incubated with the primary antibody (anti HIF1α, 1:500, Abcam) overnight at 4 °C. The next day, after washing with PBS, the membrane was incubated with horseradish peroxidase-labelled anti-rabbit secondary antibody (1:2000, Abcam) for 1 h at room temperature and thereafter washed three times with PBS. The final detection was performed with enhanced colorimetric Western blot visualization reagents using the DAB Enhanced Liquid Substrate System for Immunochemistry (Sigma-Aldrich). The results were documented using Molecular Imager Gel Doc TMXR+ (BioRad, Hercules, CA, USA). Loading differences were normalized with β-actin antibody (Santa Cruz Biotechnology) against the housekeeping control of β-actin.

### 4.13. Statistical Analysis

Statistical analysis was performed using the Statistica program, version 13 (StatSoft, Kraków, Poland). The data was analyzed for normal distribution using Kolmogorov–Smirnov and W Shapiro Wilk tests. The statistical differences or similarities between the cells that were growing in normoxic and physioxic conditions were studied using Student’s *t*-test. If not stated otherwise, values given represent means ± SD. In all analyses, *p*-values < 0.05 were considered to be statistically significant.

## 5. Conclusions

In conclusion, our study describes for the first time the influence of physiological oxygen on cell-to-cell and cell-to-extracellular matrix adhesion of single lymphoma cell. Based on this investigation, we suggest that the single cell adhesion assays performed e.g., by optical tweezers allows for much more precise determination of changes in adhesion than population assays. We also emphasize that the characterization of adhesion properties of individual cell can help in precise and repeatable monitoring of adhesion changes after the cells were treated by external stimuli, including drugs or environmental stressors.

## 6. Patents

This work resulted in two patent applications: 1. Compact hypoxic chamber compatible with optical tweezers, no. P.424002, Polish Patent Office, 21 December 2017; 2. New diagnostic tool for non-Hodgkin’s Lymphomas, no. P.423266, Polish Patent Office, 25 October 2017.

## Figures and Tables

**Table 1:** Table showing the average contact time required for the formation of adhesion between diffuse large B-cell lymphoma (DLBCL) cells and mesenchymal stromal cells (MSCs)/Matrigel in optical tweezers in normoxia. ATCC-American Type Culture Collection, DSMZ-German Collection of Microorganisms and Cell Cultures, N-number of cells measured in tree independent experiments, OT-optical tweezers, SD-standard deviation.

Cell Line (Supplier)	Type	Time-Dependent Adhesion to MSCs [s] ± SD	*N*	Time-Dependent Adhesion to Matrigel [s] ± SD	*N*	Adhesion to Matrigel vs. MSC Ratio	Adhesion Properties in OT
Ri-1 (DSMZ)	ABC	15.5 ± 8.4	81	67.5 ± 25.5	75	4.4	high/medium
U-2904 (DSMZ)	ABC	103.5 ± 34.2	77	291.2 ± 41.8	75	2.8	low
Pfeiffer (ATCC)	GCB	55.9 ± 19.4	80	89.9 ± 26.9	73	1.6	medium
Toledo (ATCC)	GCB	132.9 ± 48.8	81	223.6 ± 58.7	73	1.7	low
U-2932 (DSMZ)	GCB	34.6 ± 11.6	79	50.7 ± 16.8	74	1.5	high
SUDHL-10 (DSMZ)	GCB	19.7 ± 7.5	81	43.8 ± 14.1	79	2.2	high

**Table 2:** Fold change in time-dependent adhesion to mesenchymal stromal cells (**A**) and Matrigel (**B**) in physioxia in relations to normoxia. MSCs–mesenchymal stromal cells.

Fold Change		
Cell Line	Physioxia vs. Normoxia to MSCs	Physioxia vs. Normoxia to Matrigel
Ri-1	5.1	3.2
U2904	2.1	1.1
Pfeiffer	1.7	2.5
Toledo	2.1	1.6
U2932	3.3	2.8
SUDHL-10	4.0	2.1

## Abbreviations

ATCC	American Type Culture Collection
BS	Beamsplitter
BSA	Bovine serum albumin
CAMs	Cellular adhesion molecules
CDH2	Cadherin-2
DLBCL	Diffuse large B-cell lymphoma
DM	Dichroic mirror
DSMZ	German Collection of Microorganisms and Cell Cultures
EMC	Extracellular matrix
FBS	Fetal bovine serum
GMXY	Galvano-mirror XY scanning system
HIF1α	Hypoxia-inducible factor 1-alpha
ITGA1	Integrin alpha-1
ITGB1	Integrin beta-1
ITGB2	Integrin beta-2
ITGB7	Integrin beta-7
L	Lens
M	Mirror
MSC	Mesenchymal stromal cell
NA	Numerical aperture
OA	Optical absorbance
OCC	Oxygen controlling chamber
OT	Optical tweezers
PBS	Phosphate buffered saline
S	Spectrophotometer
SLM	Spatial light modulator
